# Ozone-Loaded Hydrogels as an Eco-Friendly Strategy to Control Phototrophic Biofilms on Cultural Heritage Surfaces

**DOI:** 10.3390/gels11110888

**Published:** 2025-11-04

**Authors:** Erica Sonaglia, Jessica Campos, Mohammad Sharbaf, Emily Schifano, Anna Candida Felici, Luciana Dini, Daniela Uccelletti, Maria Laura Santarelli

**Affiliations:** 1 Department of Chemical Engineering Materials and Environment, Sapienza University of Rome, Via Eudossiana 18, 00184 Rome, Italy; erica.sonaglia@uniroma1.it (E.S.); jessica.campos@uniroma1.it (J.C.); mohammad.sharbaf@uniroma1.it (M.S.); 2 Department of Biology and Biotechnologies “C. Darwin”, Sapienza University of Rome, P.Le Aldo Moro 5, 00185 Rome, Italy; emily.schifano@uniroma1.it (E.S.); luciana.dini@uniroma1.it (L.D.); daniela.uccelletti@uniroma1.it (D.U.); 3 Department of Basic and Applied Sciences for Engineering, Sapienza University of Rome, Via Antonio Scarpa 16, 00161 Rome, Italy; annac.felici@uniroma1.it

**Keywords:** antimicrobial hydrogel, biodeterioration, sustainable conservation, green biofilm

## Abstract

Biodeterioration represents a major threat to cultural heritage, as microbial colonization can cause both esthetic and structural damage. The use of conventional chemical biocides raises concerns due to environmental and health risks, potential substrate deterioration, and the emergence of resistant strains. In this study, an ozone-loaded bacterial cellulose (OBC) hydrogel was investigated as an eco-friendly, broad-spectrum antimicrobial treatment in the case study of the Cryptoporticus of the Baths of Trajan (Rome, Italy), a hypogean archeological site where some structures are severely affected by phototrophic biofilms. Microorganisms isolated from a colonized wall were employed in laboratory assays. OBC hydrogel exhibited strong antimicrobial activity, with >90% bacterial mortality within 10 min, complete inhibition of fungal spore germination after 24 h, and a marked reduction in microalgal chlorophyll fluorescence comparable to heat-killed controls. Furthermore, tests on Carrara marble and brick specimens artificially contaminated with microalgae confirmed the removal of green staining, restoring surface chromatic parameters (ΔE* < 5) comparable to those obtained with a commercial biocide. In situ applications demonstrated significant suppression of green biofilm for at least two months. These findings support OBC hydrogel as a sustainable, effective, and non-toxic alternative to conventional biocides for controlling microbial and microalgal colonization on cultural heritage surfaces.

## 1. Introduction

The biodeterioration of cultural heritage materials represents a great conservation challenge, particularly due to microbial colonization that leads to the formation of biofilms. These complex communities, composed of photoautotrophic and heterotrophic microorganisms, can adhere to cultural surfaces and contribute to both esthetic and structural damage over time [1].

Traditionally, conservation efforts have relied on the use of chemical biocides to prevent the growth and spread of these organisms. However, such treatments raise substantial concerns, including environmental contamination, health hazards for those in contact with the object, substrate deterioration, and the emergence of resistant microbial strains [2,3,4]. In response, there is growing interest in the development of green biocides. They are naturally derived, environmentally friendly alternatives that offer antimicrobial efficacy while reducing ecological and health risks [5].

In this context, an innovative ozone-loaded bacterial cellulose (OBC) hydrogel was developed to be used as a green biocide. It demonstrated significant antimicrobial performance in laboratory studies against biodeteriogenic microorganisms, without leaving any residues on the treated areas [6]. In addition to this, it can be produced at low costs when compared to traditional biocides. Bacterial cellulose (BC) is a biodegradable, non-toxic, and renewable biopolymer that naturally occurs in hydrogel form. Its production can be sustainable and its application in conservation is gaining momentum due to its favorable mechanical and physicochemical properties [6,7]. In this formulation, ozone, known for its broad-spectrum antimicrobial activity [8] and clean degradation into oxygen, acts as the active agent.

The present work further extends the antimicrobial properties of the OBC hydrogel versus green biofilm present in the real case study of the Cryptoporticus of the Baths of Trajan, a site of historical and architectural significance located on the Oppian Hill in Rome. Constructed in the early 2nd century AD under Emperor Trajan, the Cryptoporticus forms part of a vast underground corridor supporting the monumental bath complex [9]. Ongoing excavations and studies have brought to light multiple historical layers within the site. The Cryptoporticus lies beneath a well-preserved exedra, often hypothesized to have functioned as a library, and features a vaulted corridor over 8 m wide. Archeological findings in this area have included a 19th-century Napoleonic powder magazine, a frescoed wall section depicting a fortified city known as the “Painted City” [10], and fragments of a finely executed parietal mosaic, showcasing chiaroscuro modeling and decorative architectural elements [11]. These discoveries highlight the remarkable artistic and historical significance of the site. The hypogean nature of the site, characterized by high relative humidity, stable temperatures, limited ventilation, and the penetration of natural light, creates ideal conditions for microbial colonization, especially of green biofilms, which are clearly visible on various surfaces throughout the archeological site.

This study deeply investigates the practical potential of OBC hydrogels as a sustainable biocidal tool for the treatment of biofilms on cultural heritage surfaces, through the assessment of their antimicrobial activity against photoautotrophic and heterotrophic microorganisms isolated from the site, as well as through the in situ effectiveness evaluation.

## 2. Results and Discussion

### 2.1. Cultivable Microorganisms Collected from the Cryptoporticus of the Baths of Trajan

Samples collected from a non-contaminated wall (C) and from a wall covered by green biofilm (S) in the Cryptoporticus of the Baths of Trajan revealed a diverse community of cultivable microorganisms. Among heterotrophic microorganisms, three bacterial and two fungal strains were isolated from both samples. Figure S1 shows the isolated microorganisms on Petri plates. DNA extraction, amplification, sequencing, and comparison with database sequences were performed on the isolated bacteria and fungi for their identification. The bacteria were identified as *Burkholderia* sp., *Mucilaginibacter* sp. and *Actinacidiphila bryophytorum*. Bacteria belonging to the *Burkholderia* genus have already been reported colonizing cultural heritage stone surfaces in hypogean environments [12], but also on canvas [13]. *Mucilaginibacter* bacteria have also been previously characterized in the soil of several archeological sites [14,15] or on stone cultural heritage surfaces, also in association with green biofilm [16,17]. These microorganisms can strongly interact with heritage substrates, leading to both esthetic and physico-chemical deterioration. Fungal strains identified are *Aspergillus sydowii* and *Cladosporium herbarum*. Fungal strains belonging to the *Aspergillus* and *Cladosporium* genera have been previously documented in Roman catacombs [18] and other hypogean archeological sites [19,20,21]. The photoautotrophic microorganisms were detected only in the sample S. They were identified by morphological comparison as microalgae belonging to the *Chlorella* and *Klebsormidium* genera (Figure S2). These genera are frequently observed colonizing cultural heritage sites globally [22,23,24]. Considering the taxonomic and functional diversity of photoautotrophic and heterotrophic microorganisms inhabiting the selected wall in the Cryptoporticus of the Baths of Trajan, a broad-spectrum biocidal treatment is required to ensure effective control and management.

### 2.2. Antimicrobial Activity of OBC Against Bacteria and Fungi

**Figure 1:**
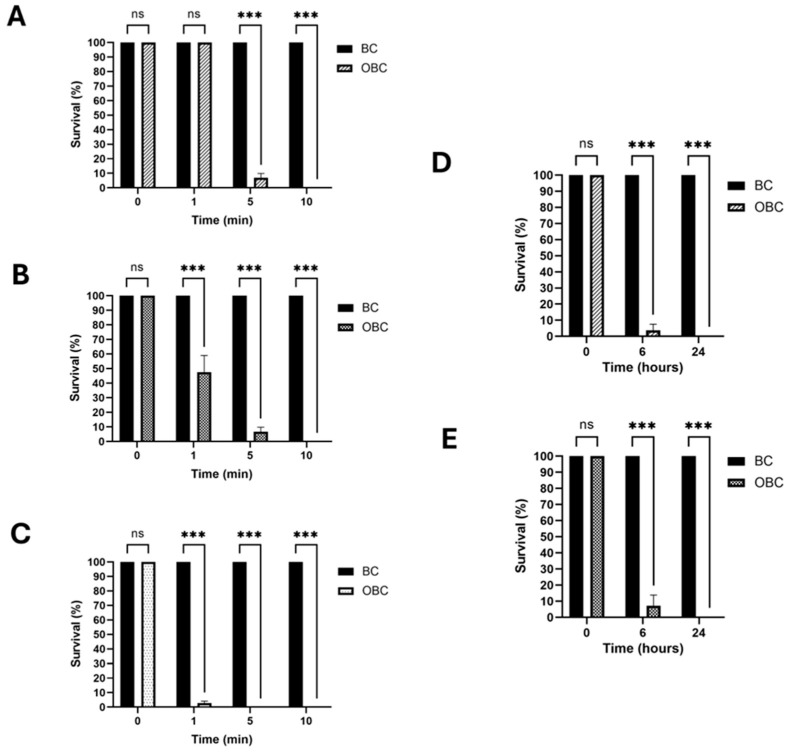
Impact of OBC hydrogels on bacterial and fungal viability. *Burkholderia* sp. (**A**), *Mucilaginibacter* sp. (**B**) and *Actinacidiphila bryophytorum* (**C**) were subjected to treatment with BC (control) and OBC hydrogels for different times, as indicated. Bacterial survival was evaluated using colony-forming unit (CFU) counting analysis. The germination capability of *Aspergillus sydowii* (**D**) and *Cladosporium herbarum* (**E**) spores was assessed after treatment with BC (control) or OBC hydrogels by CFU counting after different treatment times. Data are presented as mean  ±  SD. Statistical analysis was carried out using a one-way ANOVA with the Bonferroni post-test (ns *p*  > 0.05 and *** *p*  <  0.001 compared to the BC sample).

Laboratory-prepared OBC hydrogels containing 0.641 mmol/g of ozone were used to assess the biocidal effect. To evaluate the antibacterial efficacy, a colony-forming unit (CFU) counting analysis was conducted in a liquid assay using the isolated bacterial species. Following a 5 min treatment, the mortality of *Burkholderia* sp. exceeded 90%, with a complete suppression of viability after 10 min (Figure 1A). A similar level of suppression was obtained for *Mucilaginibacter* sp. strain (Figure 1B). For *Actinacidiphila bryophytorum*, the treatment produced approximately 95% mortality after only one minute, reaching complete suppression of viability after 5 min (Figure 1C). The treatment is effective on both Gram-positive (*Actinacidiphila bryophytorum*) and Gram-negative (*Burkholderia* sp., *Mucilaginibacter* sp.) bacterial strains, demonstrating the high-spectrum antibacterial activity. Differences in antimicrobial efficacy among bacterial strains can be ascribed to variations in the cell wall composition, which influences susceptibility to ozone [6,25,26].

The anti-sporogenic effect against the two fungal species was evaluated by assessing the germination ability of spores by the CFU counting method after treatment with OBC hydrogels in a liquid assay. Specifically, for *Aspergillus sydowii*, a mortality rate exceeding 94% was obtained after a 6 h-treatment (Figure 1D), reaching a complete suppression after a 24 h-treatment. A similar trend was observed for *Cladosporium herbarum* (Figure 1E). Fungal spores, with their robust protective outer protein layer and relatively impermeable inner membrane, exhibit high resistance to chemicals, necessitating extended treatment time, as already assessed in previous reports [27,28]. The variability of the characterized biodeteriogens can highlight the challenge in the treatment of the complex biofilm; however, ozone’s broad-spectrum nature makes it a highly effective biocide with fully antimicrobial activity.

### 2.3. Antimicrobial Activity of OBC Against Microalgae in Liquid Assay

**Figure 2:**
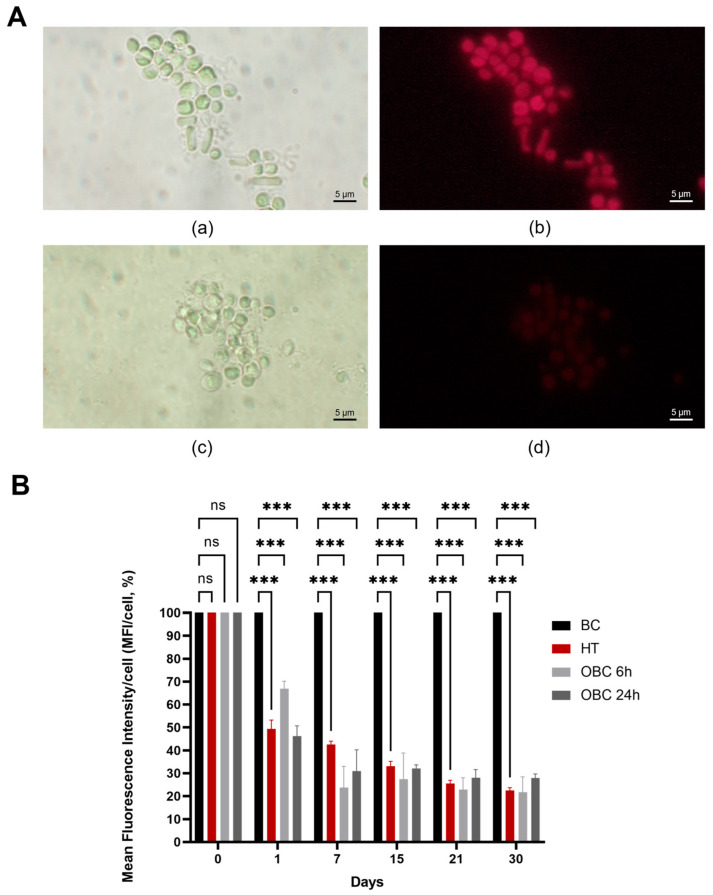
Impact of OBC hydrogels on microalgal viability. (**A**) Fluorescence micrographs of microalgae subjected to treatment with BC (**a**,**b**) or with OBC (**c**,**d**) hydrogels for 24 h, observed after 7 days. Images were collected using the bright field (**a**,**c**) and under red channel fluorescent light (**b**,**d**). (**B**) The mean fluorescence intensity per cell (MFI/cell) was expressed as a percentage relative to the negative control at specific time points (days) following the treatment with heat (HT) or with OBC hydrogels for 6 h (OBC 6 h) or 24 h (OBC 24 h). Data are presented as mean  ±  SD. Statistical analysis was carried out using a one-way ANOVA with the Bonferroni post-test (ns *p*  > 0.05 and *** *p*  <  0.001 compared to the BC sample).

The antimicrobial potential of OBC was evaluated against microalgae collected from the Cryptoporticus of the Baths of Trajan in liquid assays. Two treatment durations, 6 and 24 h, were tested. Chlorophyll fluorescence measurements were employed to assess microalgal viability. In Figure 2A, the fluorescence of microalgae subjected to 24 h-treatment with BC (a, b) or with OBC (c, d) hydrogels is reported after 7 days since the treatments. The strong reduction in the autofluorescence of the OBC-treated microalgae is evident, in contrast with the BC-treated control, where this feature is still present. The reduction in fluorescence is due to the degradation of chlorophyll into non-fluorescent derivatives [29]. The results of fluorescence quantification are presented in Figure 2B following the treatment, with values expressed as a percentage relative to the negative control treated with BC. Heat treatment (HT), used as a positive control, demonstrated that microalgal cells retain residual chlorophyll fluorescence even after death, the values ranging from 50% to 20% with respect to the untreated cells along the analyzed period. A similar behavior was observed for the OBC-treated cells. The results are consistent with studies on the fluorescence of phototrophic microorganisms treated with other biocides [2,30,31].

### 2.4. Antimicrobial Activity of OBC on Stone Specimens Contaminated by Microalgae

**Figure 3:**
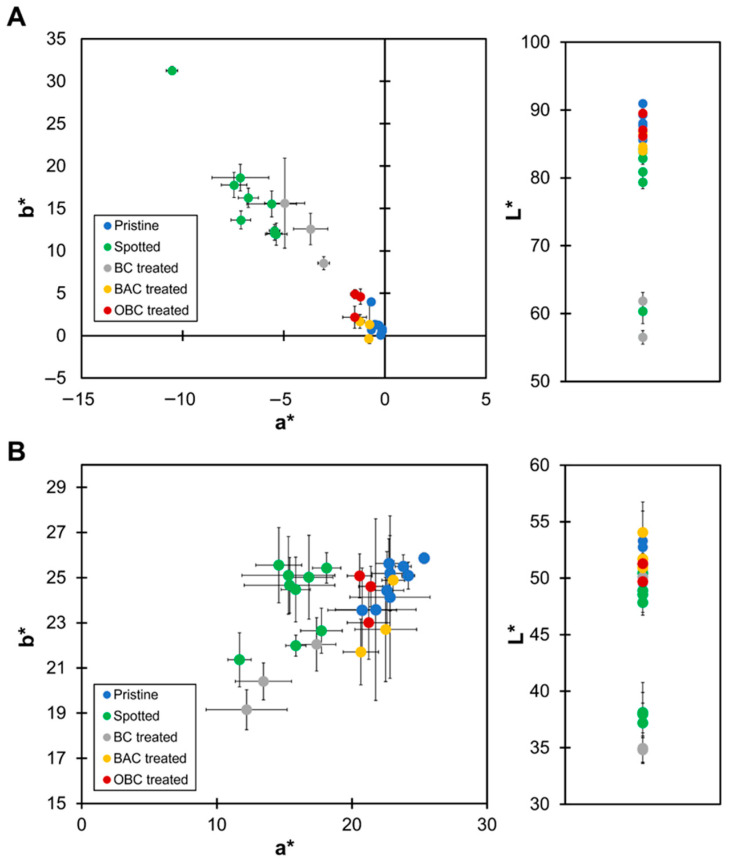
Colorimetric analysis of marble (**A**) and brick (**B**) specimens. Chromatic parameters L*, a*, and b* of the pristine surfaces (Pristine, blue), the contaminated surfaces (Spotted, green) and after the treatment with BC (gray), BAC (orange), or OBC (red).

Colorimetric analysis is a well-established method for assessing the presence and vitality of photoautotrophic microorganisms on stone surfaces [32,33]. Colorimetric changes in marble and brick specimens inoculated with microalgae were quantitatively assessed using colorimetry to evaluate the efficacy of OBC treatment in restoring the esthetic appearance of colonized surfaces. Measurements were conducted on pristine specimens (Pristine) and on microalgae-colonized surfaces (Spotted). The specimens were subjected to different treatments, BC, OBC and benzalkonium chloride (BAC), as a positive control. Results are summarized in Figure 3. For marble samples (Figure 3A), microalgae colonization resulted in substantial shifts in all three chromatic parameters. Spotted samples exhibited a decrease in L* values (darker appearance) and in a* values (greener tones), and increased b* values (yellower tones) relative to pristine surfaces, confirming the formation of a green biofilm. These findings align with previous studies demonstrating that microalgal colonization leads to significant discoloration of stone surfaces [34]. Treatment with the BC hydrogel produced only marginal changes in colorimetric parameters, with L*, a*, and b* values remaining close to those of the Spotted samples, confirming the absence of biocidal activity. In contrast, the OBC-treated specimens showed a pronounced restoration of the original color, as indicated by L*, a*, and b* values approaching those of the pristine samples. The same result was obtained for specimens treated with BAC. For brick samples (Figure 3B), an identical trend was observed, with greater color variability due to the multi-mineral composition of the material, also reflected in pristine samples. The results suggest that OBC hydrogel can achieve color restoration on both lithotypes comparable to conventional biocidal treatments, while offering a more environmentally friendly approach.

The total color change (ΔE*) mean values, calculated as the difference between the color coordinates of the pristine specimens and those measured after BC, BAC, and OBC treatments, respectively, provide a quantitative assessment of the cleaning efficacy. Indeed, this parameter is widely recognized as a reliable indicator of surface alteration in the conservation of stone cultural heritage materials [35]. As shown in Table 1, the treatment with pristine BC resulted in the highest ΔE* values, indicating a pronounced chromatic alteration due to biological colonization. The pristine BC formulation lacks antimicrobial activity and therefore cannot inhibit microbial growth or promote surface cleaning. Consequently, the observed color change reflects the accumulation of biofilm on the treated surface, in agreement with previous studies reporting ΔE* values varying from 15 to 43 for lithotypes affected by phototrophic biofilm [36,37]. After treatment, a significant decrease in ΔE* was observed for marble and brick specimens for both the commercial biocide (BAC) and the OBC hydrogel. These results demonstrate that the OBC hydrogel exhibited a cleaning performance comparable to that of the commercial biocide, maintaining ΔE* <  5, indicative of successful cleaning [38,39]. The obtained data are consistent with the literature on natural hydrogels used against phototrophic biofilms on different lithotypes [36,40,41]. In contrast to alginate-based hydrogels containing chlorine-releasing oxidants or essential oils, the OBC hydrogel reduces the risk of polymeric and biocidal residues on stone surfaces.

### 2.5. In Situ Treatment on the Contaminated Wall in the Archeological Site of the Cryptoporticus of Baths of Trajan

**Figure 4:**
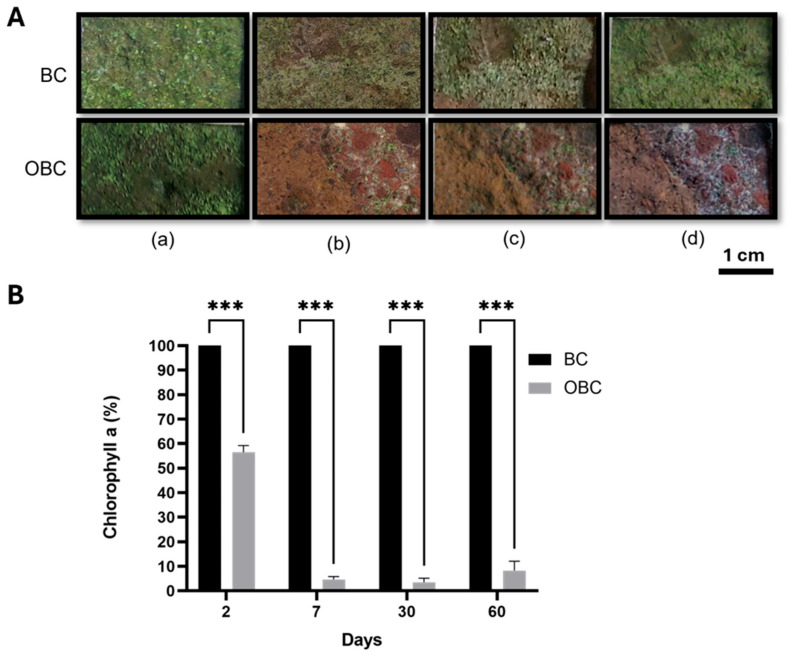
Impact of OBC hydrogels on green biofilm on the colonized wall in the archeological site of the Cryptoporticus of the Baths of Trajan. (**A**) Photographs of the areas treated with BC or OBC taken before (**a**) and 7 days (**b**), 30 days (**c**) and 60 days (**d**) after the treatments. (**B**) Chlorophyll *a* concentration expressed as a percentage relative to BC in samples collected at specific time points (days) following the in situ treatment. Data are presented as mean  ±  SD. Statistical analysis was carried out using a one-way ANOVA with the Bonferroni post-test (*** *p*  <  0.001 compared to the BC sample).

The in situ application of the OBC hydrogel on the contaminated wall at the Cryptoporticus of the Baths of Trajan was performed during the summer with the environmental temperature reaching its highest values (Figure S3). The treatment yielded compelling evidence of its effectiveness against phototrophic biofilms. Visual examination (Figure 4A) revealed that the control area treated with pristine BC hydrogel showed no remarkable changes (Figure 4A, upper row) In contrast, the OBC-treated areas exhibited an almost complete disappearance of the green biofilm, revealing the substrate underneath (Figure 4A, lower row). Similar visual modifications have been reported in other in situ biocide applications on green biofilms on different lithotypes [31,42]. This effect was sustained throughout the 60-day monitoring period, indicating successful biocide action and inhibition of photoautotrophic microorganism regrowth.

Chlorophyll *a* extraction and quantification is an attested method to evaluate colonization by phototrophic microorganisms [34,43]. Chlorophyll *a* quantification (Figure 4B) was performed on samples collected after 2, 7, 30 and 60 days. Two days post-treatment, chlorophyll *a* concentration in the OBC-treated area was 56.5% relative to the control area. However, the mean chlorophyll fluorescence intensity per cell (MFI/cell) of this sample resulted in 25.8 ± 8.8% respect to the control. This behavior suggests an early impairment of photosynthetic efficiency, likely due to oxidative stress induced by the OBC. By seven days, the chlorophyll *a* concentration decreased to 4.6%, reflected also by the fluorescence of 22.7 ± 2.5%. Similar values in chlorophyll *a* quantification were observed after 30- and 60 days post-treatment (5.6% and 8.3%, respectively), while the values of fluorescence were 28.9 ± 3.0% and 31.0 ± 2.9%, respectively. Those results are in line with the value obtained for heat-treated microalgae. This decline directly indicates the OBC hydrogel’s success in inhibiting algal vitality by damaging the photosynthetic apparatus, aligning with findings from studies using other biocides [30,44]. Furthermore, no recolonization was observed two months after treatment, demonstrating the remarkable effectiveness of the OBC hydrogel under the tested environmental conditions.

## 3. Conclusions

This study demonstrated the strong antimicrobial efficacy of the ozone-loaded bacterial cellulose (OBC) hydrogel as a sustainable biocide for cultural heritage conservation. The OBC hydrogel showed rapid and broad-spectrum activity against bacteria, fungi, and microalgae isolated from a wall contaminated with phototrophic biofilm sited at the hypogean archeological site of the Cryptoporticus of the Baths of Trajan, achieving full suppression of microbial viability and chlorophyll degradation. Colorimetric analyses confirmed effective cleaning of artificially contaminated marble and brick specimens with microalgae, restoring their original chromatic parameters (ΔE*  <  5), comparable to conventional chemical biocides but without their environmental drawbacks. The in situ application on the selected wall at the archeological site further validated these results, with significant and long-lasting removal of green biofilms and no recolonization observed after 60 days. Overall, the OBC hydrogel provides an eco-friendly, biodegradable, low-cost and effective alternative to traditional biocides, representing a promising advancement in sustainable and simple conservation strategies for cultural heritage surfaces.

## 4. Materials and Methods

### 4.1. Materials

Culture media were purchased from Biosigma, Cona, Italy. The bacterial DNA extraction kit was purchased by Qiagen, SIAL, Rome, Italy. Chemical reagents were obtained from Sigma-Aldrich, Burlington, MA, USA.

### 4.2. Microorganisms’ Collection, Isolation and Cultivation from the Cryptoporticus of the Baths of Trajan

**Figure 5:**
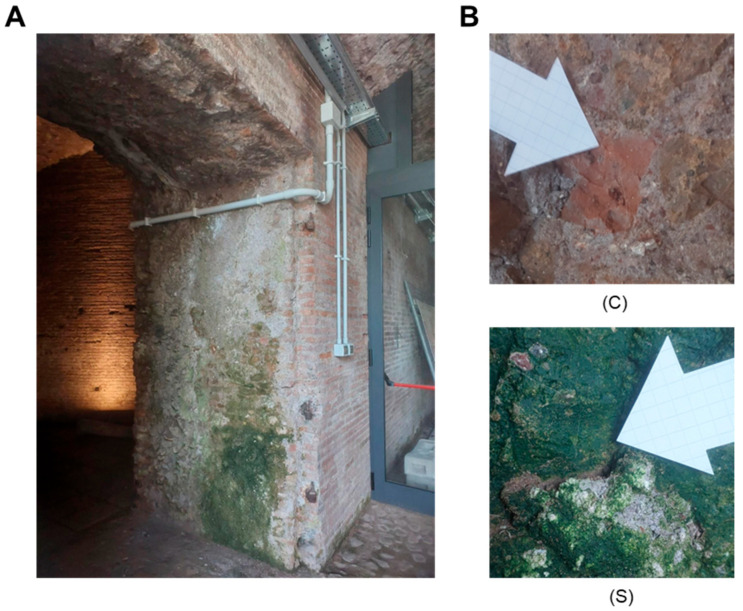
Photograph of the extensively colonized wall in Cryptoporticus of the Baths of Trajan, Rome (**A**) and sampling areas (**B**), named S and C (control sample).

Autotrophic and heterotrophic microorganisms were collected from a wall extensively colonized by a green biofilm at the archeological site of the Cryptoporticus of the Baths of Trajan on the Oppian Hill (Rome, Italy) (Figure 5A). Sampling zones (Figure 5B) were selected, one (S) belonging to a dark green part, and another (C) from a zone free from green biofilm, to work as a control. Samples were collected by scraping the wall with a sterile scalpel, suspended in 3 mL of H_2_O_dd_, and used immediately. Cultivable bacterial and fungal strains were isolated as described in [45]. Samples were plated on Nutrient Broth (NB) for bacteria and on solid Sabouraud Dextrose Agar (SDA) for fungi. Cultures were incubated under aerobic conditions at 28 °C for two days, and morphologically distinct colonies were selected and streaked on new plates for isolation. For photoautotrophic microorganisms, serial dilutions of the collected material were plated on solid Blue Green-11 (BG11). Plates were maintained in a semi-closed container with high humidity at room temperature under natural light. Single colonies with distinct morphologies were subsequently streaked on fresh plates.

### 4.3. Microorganisms Identification

Bacterial DNA was extracted and amplified according to [45]. Fungal DNA was extracted according to [46]. The DNA was treated with RNAse, re-suspended in 30 μL of Tris–EDTA buffer and stored at −20 °C for further processing. Aliquots of bacterial and fungal DNA were used for PCR amplification. For bacteria, a region of approximately 1400 bp from the 16S rRNA gene was amplified using the primers F8 (5′-AGAGTTTGATCCTGGCTCAG-3′) and R1492 (5′-GGTTACCTTGTTACGACTT-3′). For fungal strains, a region of about 600 bp from the 18S rRNA gene was amplified using the primers ITS4 (5′-TCCTCCGCTTATTGATATGC-3′) and ITS5 (5′-GGAAGTAAAAGTCGAACAAGG-3′). All PCR reactions were performed utilizing the Taq DNA polymerase from Accuzyme DNA Polymerase (Aurogene, Rome, Italy). The bacterial and fungal amplified regions were sequenced at BMR Genomics (Padova, Italy) and the obtained sequences were analyzed with the BLAST+ 2.17.0 database. Microalgae genera were morphologically identified by observation using an inverted fluorescence microscope (Axiovert 25, Carl Zeiss, Oberkochen, Germany) at 100× magnification based on cell shape and size [47].

### 4.4. Ozone-Loaded Bacterial Cellulose (OBC) Hydrogel Production

Bacterial cellulose (BC) hydrogel was sourced from an Italian brewer (Meda, MB, Italy) as a by-product of kombucha production. Samples of approximately 10 cm × 10 cm × 0.5 cm were purified as described in [48]. Ozone-loaded bacterial cellulose (OBC) hydrogels with a water holding capacity (WHC) of 152 g/g and a tensile strength of 37 MPa (dried sample) were obtained as described in [6]. For laboratory experiments, a given amount of 1 cm × 1 cm BC hydrogel samples was placed in 20 mL H_2_O_dd_ and treated by ozone bubbling for 24 h. For the in situ treatment, OBC hydrogels of 3 cm × 3 cm size were produced. Ozone was produced by an OZONIS STERIL 250 series ozone generator (Sepra S.R.L., Cesano Maderno, Italy), with a capacity of 5.2 mmol/h and a constant gas flow rate of 1.5 L/min. The ozone concentration (mmol/g) in OBC hydrogels was quantified by the DPD (N, N-diethyl-p-phenylenediamine) method described in [6]. The ozone concentration in the hydrogels resulted in 0.641 ± 0.083 mmol/g.

### 4.5. Antimicrobial Activity Assessment Against Bacteria and Fungi

The OBC antibacterial activity against bacteria and fungi isolated from the Cryptoporticus of the Baths of Trajan was evaluated. The activity against bacteria and fungi was assessed as described in [6]. As controls, bacterial cells and fungal spores were treated with BC pristine hydrogel and subjected to the same protocol. The experiments were conducted in triplicate and repeated a minimum of three times.

### 4.6. Antimicrobial Activity Evaluation Against Microalgae in Liquid Assay

To estimate antimicrobial potential, 1 mL of 2.7 × 10^7^ cells/mL mixed microalgae suspensions, determined by counting using a hemocytometer, was used. Microalgae were treated by a 1 cm × 1 cm OBC hydrogel for 6 or 24 h. As a negative control, one sample was treated with a pristine BC hydrogel. As a positive control, one sample was heat-treated at 65 °C for 10 min to obtain the suppression of microalgal vitality, as reported in [49]. Afterwards, the hydrogels were retrieved, and all samples were kept at approximately 25 °C with natural lighting and used for fluorescence measurements. Aliquots of microalgae suspensions were taken before and after 1, 7, 15, 21, and 30 days of the treatment. Chlorophyll fluorescence was observed as described in [30], using a HE Rhodamine filter (Em: 540–552/567–647 nm) on an inverted fluorescence microscope (Axiovert 25, Carl Zeiss, Italy) equipped with a digital camera (Axiocam 208 color, Carl Zeiss, Italy) at 100× magnification. Images were captured using the software Zen 3.1, with an exposure time of 400 ms. Mean chlorophyll fluorescence intensity per cell (MFI/cell) was obtained using the software ImageJ v. 1.54g. The results are presented as a percentage related to the negative control at the examination time.

### 4.7. Antimicrobial Activity Evaluation Against Microalgae on Stones

Specimens measuring approximately 5 cm × 5 cm × 2 cm of UV-sterilized white Carrara marble and brick were used. On each specimen, an area of 1 cm × 1 cm was inoculated with 90 μL of microalgae suspension (5.1 × 10^7^ cells/mL). The specimens were then placed in an incubation chamber having approximately 100% RH, at approximately 25 °C and natural lighting for one month, until a green staining of the surface was obtained for all specimens. The antimicrobial treatment was conducted in two applications. The treatment was executed by placing a 1 cm × 1 cm sample of OBC hydrogel on the inoculated area of the specimens and covered with sterile plastic film to minimize water evaporation. After 24 h, the treatment was repeated using a fresh OBC hydrogel. As a negative control, the same procedure was employed using the pristine BC hydrogel. As a positive control, 30 μL of a 3% *v/v* benzalkonium chloride (Antares, San Lazzaro di Savena (BO), Italy) solution was spread on the contaminated area as described in [34]. For each material type, treatments and controls were conducted in triplicate. Colorimetric measurements on the specimens were performed to assess the antimicrobial power of OBC hydrogels against microalgae. Analysis was performed using an AvaSpec spectrophotometer (Avantes, Apeldoorn, The Netherlands) equipped with an Avantes HL-2000 FHSA halogen lamp. For each specimen, five measurements were conducted on the area in the pristine state (Blank), after staining (Spotted), and after the respective treatment. The obtained coordinates L*, a*, and b* in the CIELab colorspace, describing the lightness, the red–green, and the yellow–blue components, respectively, were obtained for each specimen. The total color change (ΔE*), referring to the average values of the cleaned samples compared to the pristine ones, was calculated according to Equation (1).

ΔE* = [(ΔL*)^2^ + (Δa*)^2^ + (Δb*)^2^]^1/2^ (1)

### 4.8. In Situ Treatment

The in situ application of OBC hydrogel was executed on the contaminated wall sited in the archeological site of the Cryptoporticus of the Baths of Trajan in Rome (Italy). A 3 cm × 3 cm OBC hydrogel was applied by tweezers on the wall surface, and then covered with a plastic film to minimize evaporation. After 24 h, the hydrogel was replaced with a fresh one for another 24 h. As a control, an adjacent area was treated with the pristine BC hydrogel. To evaluate antimicrobial efficiency, samples from treated and control areas were collected after 2, 7, 30 and 60 days from the treatment by gently scraping a 1.0 × 1.0 cm^2^ surface cm with a sterile scalpel. Each sample was suspended in 0.2 mL and analyzed. Chlorophyll extraction was executed as described in [50], with some modifications. Each sample was split and each aliquot was resuspended in 100 μL of cold 96% ethanol (Panreac, Barcelona, Spain) and subjected to 3 pulses of 1 min-sonication, each followed by a 30 s cooling period on ice. Afterward, additional ethanol was added until 1 mL volume, and the mixture was centrifuged at 10,000 rpm for 10 min. The supernatant was then collected and used to measure absorbance at 665 nm, 649 nm, and 750 nm using a LLG-uniSPEC 2 spectrophotometer (LLG Labware, Meckenheim, Germany), with pure ethanol as blank. Chlorophyll *a* (C*a*) concentration was calculated using Equation (2) according to [50], where C*a* represents the concentration (µg/mL), and A665 and A649 correspond to the absorbance values at 665 nm and 649 nm, respectively.

C*a* = 13.95 × A_665_ − 6.88 × A_649_ (2)

Each absorbance reading was corrected by subtracting the absorbance measured at 750 nm to account for background interference. The concentration was then converted in mg/cm^2^ and expressed as a percentage with respect to the control sample collected at the same time slot. To further validate the obtained results, fluorescence measurements were conducted on the collected samples prior to extraction, as previously described.

## Figures and Tables

**Table 1:** Total color change (ΔE*) between pristine and treated surfaces of marble and brick specimens after BC, BAC, and OBC treatments. Data are mean values ± standard deviation (*N* = 3).

Treatment	∆E* Marble	∆E* Brick
BC	24.79 ± 4.66	13.99 ± 3.87
BAC	2.28 ± 1.52	4.16 ± 0.54
OBC	3.84 ± 1.01	2.96 ± 0.58

## Data Availability

The original contributions presented in this study are included in the article/Supplementary Material. Further inquiries can be directed to the corresponding author.

## Supplementary Materials

The following supporting information can be downloaded at: https://www.mdpi.com/article/10.3390/gels11110888/s1, Figure S1: Isolated microorganisms on Petri plates from the wall at the Cryptoporticus of the Baths of Trajan (Rome, Italy) archeological site. (a) Bacteria identified as *Burkholderia* sp. (B1), *Mucilaginibacter* sp. (B2) and *Actinacidiphila bryophytorum* (B3); (b) *Aspergillus sydowii*; (c) *Cladosporium herbarum*; Figure S2: Microphotographs in bright field (100× magnification) of microalgae isolated from the wall at the Cryptoporticus of the Baths of Trajan (Rome, Italy) archeological site, identified as belonging to *Klebsormidium* (a) and *Chlorella* genera (b); Figure S3: Temperature measurements recorded monthly in 2023 in the entrance erea of the Cryptoporticus of the Baths of Trajan, Rome.

## Abbreviations

The following abbreviations are used in this manuscript:

OBC	Ozone-loaded Bacterial Cellulose
BC	Bacterial Cellulose
CFU	Colony Forming Unit
HT	Heat Treatment
BAC	Benzalkonium Chloride
Tris–EDTA	Tris(hydroxymethyl)aminomethane–Ethylenediaminetetraacetic Acid
NB	Nutrient Broth
SDA	Sabouraud Dextrose Agar
BG11	Blue Green-11 Medium
RH	Relative Humidity
