# Supplementary material for: Ozone-Loaded Hydrogels as an Eco-Friendly Strategy to Control Phototrophic Biofilms on Cultural Heritage Surfaces
Source: Gels. 2025 Nov 4;11(11):888. doi: 10.3390/gels11110888 (PMC12652493; doi:10.3390/gels11110888)
Supplement: Supplementary file 1 [file gels-11-00888-s001.zip › gels-3968167-supplementary.pdf]

Ozone-Loaded Hydrogels as an Eco-Friendly Strategy to Control Phototrophic Biofilms on Cultural Heritage Surfaces

Erica Sonaglia [1], Jessica Campos [1], Mohammad Sharbaf [1], Emily Schifano [2], Anna Candida Felici [3], Luciana Dini [2], Daniela Uccelletti [2] and Maria Laura Santarelli [1,*]

[1] Department of Chemical Engineering Materials and Environment, Sapienza University of Rome, Via Eudossiana 18, 00184 Rome, Italy; erica.sonaglia@uniroma1.it (E.S.); jessica.campos@uniroma1.it (J.C.); mohammad.sharbaf@uniroma1.it (M.S.)
[2] Department of Biology and Biotechnologies "C. Darwin", Sapienza University of Rome, P.Le Aldo Moro 5, 00185 Rome, Italy; emily.schifano@uniroma1.it (E.S.); luciana.dini@uniroma1.it (L.D.); daniela.uccelletti@uniroma1.it (D.U.)
[3] Department of Basic and Applied Sciences for Engineering, Sapienza University of Rome, Via Antonio Scarpa 16, 00161 Rome, Italy; annac.felici@uniroma1.it
* Correspondence: marialaura.santarelli@uniroma1.it

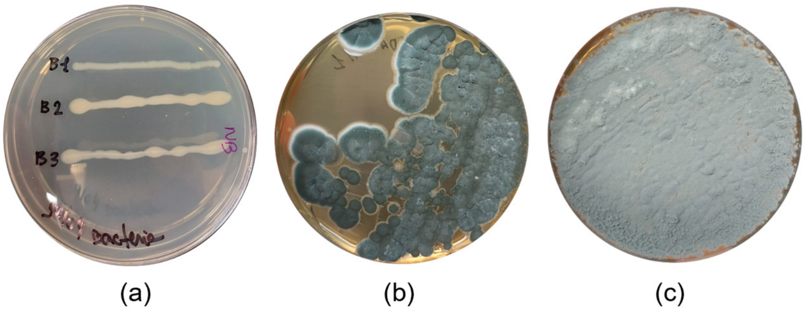

Figure S1. Isolated microorganisms on Petri plates from the wall at the Cryptoporticus of the Baths of Trajan (Rome, Italy) archaeological site. (a) Bacteria identified as *Burkholderia* sp. (B1), *Mucilaginibacter* sp. (B2) and *Actinacidiphila bryophytorum* (B3); (b) *Aspergillus sydowii*; (c) *Cladosporium herbarum.*

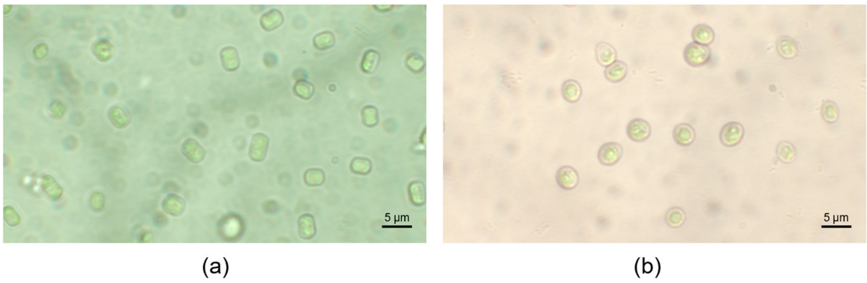

Figure S2. Microphotographs in bright field (100X magnification) of microalgae isolated from the wall at the Cryptoporticus of the Baths of Trajan (Rome, Italy) archaeological site, identified as belonging to *Klebsormidium* (a) and *Chlorella* genera (b).

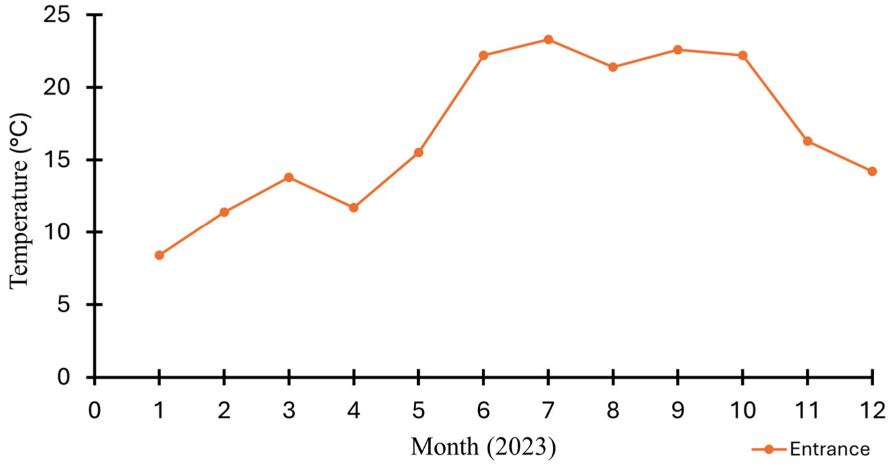

Figure S3. Temperature measurements recorded monthly in 2023 in the entrance erea of the Cryptoporticus of the Baths of Trajan, Rome.
